# Inferior Right Hepatic Vein: A Useful Anatomic Variation for Isolated Resection of Segment VIII

**DOI:** 10.1155/2013/371264

**Published:** 2013-08-19

**Authors:** Klaus Steinbrück, Reinaldo Fernandes, Giuliano Bento, Rafael Vasconcelos, Gustavo Stoduto, Thomas Auel, Lúcio F. Pacheco-Moreira

**Affiliations:** Hepatobiliary Surgery Unit, Bonsucesso Federal Hospital—Health Ministry, Av. Londres, 616, prédio 3/2° andar, 21041-030 Rio de Janeiro, RJ, Brazil

## Abstract

Anatomical resection of segment VIII (SVIII) is one of the most difficult hepatectomies to perform. Although it is the best choice of surgical treatment for tumors located at SVIII, its feasibility can be compromised when the right hepatic vein (RHV) must be resected en bloc with SVIII. Herein we describe a case of a cirrhotic patient that was submitted to segmentectomy VIII in bloc with the main trunk of the RHV, due to hepatocellular carcinoma. The resection could only be performed because a well developed inferior right hepatic vein (IRHV) was present. Anatomical variations of the liver vascularization should be used by liver surgeons to improve surgical results.

## 1. Background

Right hepatectomy is the procedure of choice for most patients with normal liver function and hepatic tumors located in the right liver, especially those in close contact to the right hepatic vein (RHV). Parenchyma-sparing resection may be required, however, in patients with impaired liver function and this is common in patients with cirrhosis and hepatocellular carcinoma (HCC).

 For tumors confined to segment VIII (SVIII) of the liver, the procedure of segmentectomy VIII offers a chance of anatomically resecting the tumor while preserving most of the liver tissue. This procedure, however, can be really challenging when the tumor is to close to the RHV.

 Up to 21% of patients have a large inferior right hepatic vein (IRHV) that drains the inferior segments of the right hemiliver [1]. The presence of this variation facilitates the isolated resection of SVIII, even when the RHV must be ligated and divided.

 Herein we describe a case of isolated segmentectomy VIII due to HCC in a cirrhotic patient, in which the presence of an IRHV enabled the section of the RHV without harming the drainage of inferior segments.

## 2. Patient and Methods

**Figure 1:**
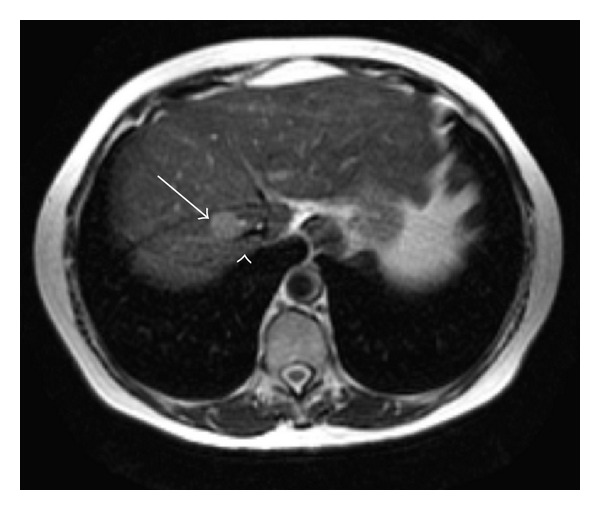
T2 weighted Magnetic Resonance image showing a tumor (arrow) compressing the RHV (arrow head).

**Figure 2:**
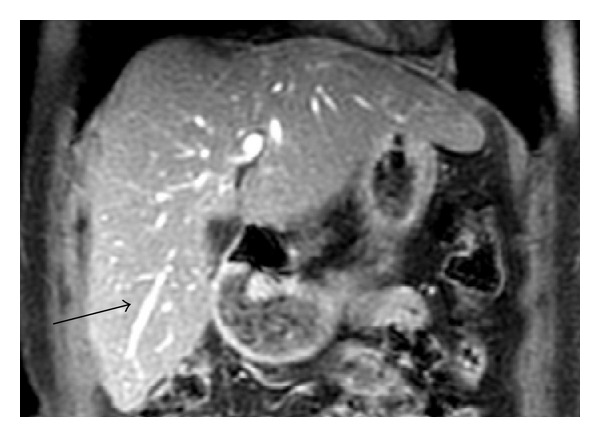
T1 weighted Magnetic Resonance image showing inferior right hepatic vein (arrow).

 A 66-year-old woman was admitted to our hospital with an abdominal ultrasonography showing a 2 cm nodule in SVIII of the liver. She had been previously diagnosed with Hepatitis B, but received no further treatment. Laboratory tests revealed AST 34 U/L, ALT 28 U/L, total bilirubin 0.8 mg/dL, INR 1.1 and albumin 4.5 g/dL, which demonstrated a minor liver disfunction. As she had no ascites or encephalopathy, she was classified as Child-Pugh's grade A. Alpha-fetoprotein level was 845 ng/mL. MRI confirmed a 2.5 tumor touching and compressing the main trunk of RHV (Figure 1) and was consistent with HCC. The exam revealed also an IRHV (Figure 2) draining segments V and VI directly into the inferior vena cava, which encourage us to perform an isolated resection of segment VIII with ligation and division of the RHV.

**Figure 3:**
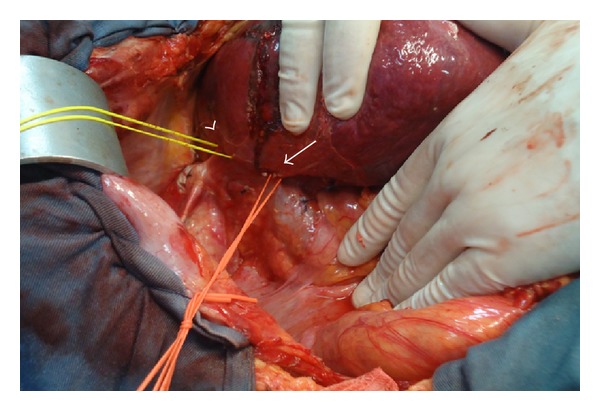
Retrohepatic inferior vena cava. RHV (arrow head) and inferior right hepatic vein (arrow) are encircled.

**Figure 4:**
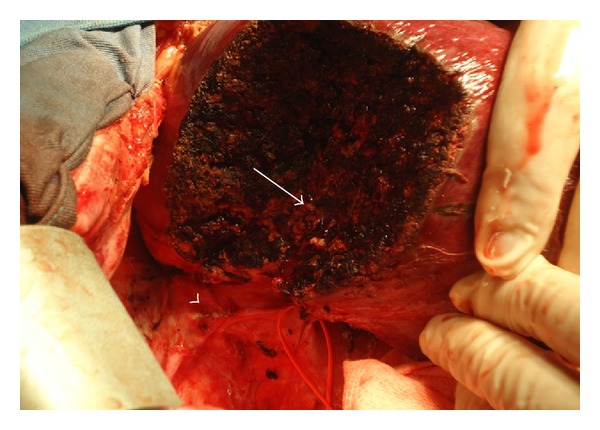
Final aspect of surgery. Divided RHV (arrow head) and portal pedicle (arrow) can be seen.

 Surgery was performed by bilateral subcostal laparotomy. Structures of the hepatic pedicle were dissected. Right portal vein (RPV) and right hepatic artery (RHA) were isolated. Retrohepatic inferior vena cava was dissected. RHV and IRHV were encircled (Figure 3). Using intraoperative ultrasonography, the tumor and portal branches for SVIII were identified and resection area was marked in the liver surface. Segmentectomy VIII was performed with kellyclasia after occlusion of RPV and RHA for 26 minutes (2 × 10 minutes and 1 × 6 minutes clamping with 10 minutes interval between them). The main trunk of the RHV was ligated and divided in the beginning of the procedure. Control of biliary leakage was obtained with injection of methylene blue through the cystic duct after cholecystectomy. Final aspect of surgery is shown in Figure 4.

 On post-operative (PO) period, AST, ALT, total bilirubin and INR raised up to 288 U/L, 254 U/L, 0.7 mg/dL and 1.62, respectively. On PO day 6, patient presented fever and abdominal pain. CT scan revealed fluid collection at right subphrenic space, which was confirmed as a biloma after percutaneous drainage. Patient was discharge on PO day 16 after 10 days of antibiotic therapy. She had no further complications and is doing well, with no signs of HCC relapse, after 18 months.

## 3. Discussion

 Patients with large or deeply located liver tumors in SVIII are normally submitted to right hepatectomy or extended right hepatectomy to guarantee an oncological resection of the tumor. However, these major resections may not be tolerated by patients with poor liver reserve, which is often seen in HCC associated to cirrhosis. As HCC tends to metastasize via the portal vein [2], resection of the liver parenchyma fed by portal venous branches bearing the tumour seems logical to eradicate potential intrahepatic metastases. Therefore, isolated segmentectomy can be performed to achieve both oncological and parenchyma-sparing resection.

 Among segmentectomies, isolated resection of SVIII is the most challenging and complex liver resections to perform. This segment is surrounded by the RHV laterally, the middle hepatic vein medially, and the anterior branch of the RPV inferiorly. Moreover, the absence of anatomic landmarks on the liver surface makes it difficult to determine the precise extent of resection. All these circumstances render an isolated resection of SVIII technically demanding, but still feasible to be done. Surgical techniques have already been described to provide an anatomical and secure resection of SVIII [3–5].

 In particular cases, as the one described here, the tumor may be in close contact to the RHV and its resection together with the tumor is necessary. For these cases, drainage of the right liver can be seriously compromised, unless a variant drainage system of the hepatic veins is present. Fang et al. [1] reported a study of variations of the hepatic veins and showed that in 39% of patients, RHV is not the only responsible for entire drainage of right hemiliver. A well-developed IRHV is presented in 21% of cases and provides drainage for segments V and VI.

 In the case herein reported, we performed the segmentectomy VIII associated to RHV resection because a IRHV was identified. If such anatomical variation was not present, this patient would have been elected for liver transplant, a much more aggressive treatment.

 Machado et al. [6] and Capussotti et al. [7], both in 2006, described 5 cases that resection of segments VII-VIII and RHV was successfully performed in patients without an IRHV. However, for the best of our knowledge, since 2006 only eight more similar cases were reported [8] and no cases of isolated resection of SVIII and RHV without an IRHV were described until now. We believe that the presence of an IRHV is still a limitation for segmentectomy VIII with RHV ligation and division. 

In conclusion, every patient eligible for a liver surgery must have his liver anatomy evaluated. Liver surgeons should be prepared to use anatomy variations in favor of their patients. Inferior right hepatic vein is a useful variation for isolated resection of SVIII, when the RHV must be sacrificed.
